# Measuring similarities between transcription factor binding sites

**DOI:** 10.1186/1471-2105-6-237

**Published:** 2005-09-28

**Authors:** Szymon M Kielbasa, Didier Gonze, Hanspeter Herzel

**Affiliations:** 1 Institute for Theoretical Biology, Humboldt University, Invalidenstraße 43, D-10115 Berlin, Germany; 2 Unité de Chronobiologie Théorique, Université Libre de Bruxelles, CP 231, Campus Plaine, Bvd du Triomphe, B-1050 Bruxelles, Belgium

## Abstract

**Background:**

Collections of transcription factor binding profiles (Transfac, Jaspar) are essential to identify regulatory elements in DNA sequences. Subsets of highly similar profiles complicate large scale analysis of transcription factor binding sites.

**Results:**

We propose to identify and group similar profiles using two independent similarity measures: *χ*^2 ^distances between position frequency matrices (PFMs) and correlation coefficients between position weight matrices (PWMs) scores.

**Conclusion:**

We show that these measures complement each other and allow to associate Jaspar and Transfac matrices. Clusters of highly similar matrices are identified and can be used to optimise the search for regulatory elements. Moreover, the application of the measures is illustrated by assigning E-box matrices of a SELEX experiment and of experimentally characterised binding sites of circadian clock genes to the Myc-Max cluster.

## Background

In order to dissect the complex machinery of transcriptional control computational tools are widely used [1]. Candidate binding sites of known transcription factors are located by consensus sequence search or binding scores calculated from position weight matrices (PWMs) [2]. These matrices are derived from position frequency matrices (PFMs) obtained by aligning binding sites for a given transcription factor. PFMs contain the observed nucleotide frequencies at each position of the alignment. A popular collection of eukaryotic PFMs is given by the Transfac database [3]. Furthermore, an open-access database, Jaspar [4], has been compiled recently.

On-line tools are available to calculate high-scoring binding sites on the basis of these matrix collections [5-7]. For a given transcription factor these programs predict many binding sites (on average every 1000 bp) implying a high excess of false positives [1]. The situation is even worse if hundreds of different binding profiles are studied in parallel leading to multiple testing issues. Often these predictions overlap as a result of similarities of transcription factor binding profiles.

First steps to overcome the flood of false positive signals are accurate predictions of promoter regions and enhancers [8-10]. Phylogenetic footprinting [11-13], correlation with gene expression data [14,15] or analysis of cooperative binding of multiple transcription factors [16] allow to reduce the amount of false positives by at least an order of magnitude. Another helpful strategy is the *a priori *reduction of the number of matrices to be considered. However, a user-defined preselection of a few matrices is highly subjective and might hide novel interactions of several transcription factors. Therefore, in this paper we combine two objective criteria to measure similarities of transcription factor binding site profiles. These measures allow to construct groups of similar profiles. Representative matrices of the groups may be chosen and constitute a reduced and unbiased list of independent profiles for searching binding sites.

Similarities in the collections of matrices may arise from several sources:

1. Identical transcription factors are represented by different matrices. This appears, e.g., due to the distinct nomenclature in Transfac and Jaspar (for example the TATA-binding protein is referred as TATA in Transfac and as TBP in Jaspar) or due to the availability of matrices obtained with different methods (see for example Transfac matrices SRF_01 and SRF_Q6) or stringency criteria (see for example AP1_Q2 and AP1_Q6).

2. Factors within one family are represented by similar matrices due to the conserved structure of DNA-binding domains [17]. For example, both ATF and CREB matrices belong to the same bZIP family and recognise the TGACGT consensus sequence.

3. There might be so far undetected similarities of different transcription factor binding sites. Such similarities can point to a possible cross-talk between different regulatory pathways (see our discussion of E-box binding sites below).

4. It might be difficult to distinguish matrices for which only a few binding sites are known.

In order to identify similar matrices we combine two similarity measures. The first one is based on the *χ*^2 ^distance of position frequencies of PFMs. The other utilizes scores from the corresponding position weight matrices (PWMs) – we calculate for a given pair of binding profiles the scores along a test DNA sequence and take the corresponding Pearson correlation coefficient as a similarity measure. Although related similarity measures have been already studied individually [15,17-21], our combined approach applied to the Transfac matrices reveals that the two selected measures capture different properties of the matrices and therefore the measures complement each other. Moreover, since for many matrices only a few experimentally verified binding sites are available we take into account these small sample sizes in both measures. The application of the measures is illustrated by mapping CLOCK-BMAL1 binding sites of circadian clock genes to the Myc-Max family.

## Implementation

### Databases

A commonly used database of experimentally verified transcription factor binding sites is Transfac [3]. The release from May 2004 provides 694 position frequency matrices (PFMs) covering vertebrates, plants, insects and fungi. Recently, a publicly available Jaspar database [4] was compiled with 108 PFMs associated mainly to vertebrates. For our large-scale statistical analysis we discarded all matrices with inconsistencies, for example matrices, where the number of sites aligned to construct the matrix (sample size) could not be determined. Furthermore, we excluded rather poor matrices with a length below 6 bases or a sample size below 5. After these consistency checks and filtering steps we arrived at 637 different matrices for Transfac and 103 matrices for Jaspar. All the matrices can be characterized by their length, the sample size, and the information content [22] (Tab. 1).

**Table 1:** Properties of Transfac and Jaspar matrices: We removed matrices for which the sample size was normalized to 100 and no information about the actual number of samples was available, as well as matrices of length below 6 or sample size below 5.

Property	Transfac	Jaspar
Number of original matrices	694	108
Number of matrices after filtering	637	103
Min length	6	6
Max length	30	30
Median length	12	11
Min sample size	5	6
Max sample size	389	389
Median sample size	18	23
Min information content	3.6	5.7
Max information content	44.3	26.2
Median information content	12.8	11.6

### *χ*^2 ^distance *D *between position frequency matrices

**Figure 1:**
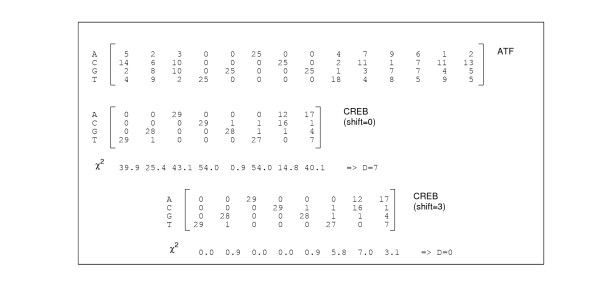
CREB versus ATF matrices: The distance *D *is computed for each possible alignment between the two matrices. For each aligned column, we calculated the *χ*^2 ^scores. *D *is then the number of *χ*^2 ^values which exceed the threshold  = 7.81 For shift= 0, the two matrices are not properly aligned, *D *= 7. For shift= 3, the two matrices are properly aligned, *D *= 0.

For each possible overlap (of at least 6 bases) of two PFMs we count the number of corresponding columns which are statistically independent. This task can be addressed by the homogeneity test using the *χ*^2 ^measure with 3 degrees of freedom. The application of PFMs for the characterization of binding sites implies that the nucleotide positions are regarded as independent. Even though statistical dependencies between positions are known [23-25] the assumption of independent positions is a rather good approximation [1,26]. In the following we denote by *f*_*b,i *_and *g*_*b,i *_the entries of the overlapping parts of the two frequency matrices to be compared. The index *i *refers to the base position along the matrices and *b *enumerates the four nucleotides A, C, G and T. The *χ*^2 ^distance at the position *i *is then given by:

χ2=∑b=A,C,G,T(Ng,ifb,i−Nf,igb,i)2Nf,iNg,i(fb,i+gb,i)
 MathType@MTEF@5@5@+=feaafiart1ev1aaatCvAUfKttLearuWrP9MDH5MBPbIqV92AaeXatLxBI9gBaebbnrfifHhDYfgasaacH8akY=wiFfYdH8Gipec8Eeeu0xXdbba9frFj0=OqFfea0dXdd9vqai=hGuQ8kuc9pgc9s8qqaq=dirpe0xb9q8qiLsFr0=vr0=vr0dc8meaabaqaciaacaGaaeqabaqabeGadaaakeaacqaHhpWydaahaaWcbeqaaiabikdaYaaakiabg2da9maaqafabaWaaSaaaeaacqGGOaakcqWGobGtdaWgaaWcbaGaem4zaCMaeiilaWIaemyAaKgabeaakiabdAgaMnaaBaaaleaacqWGIbGycqGGSaalcqWGPbqAaeqaaOGaeyOeI0IaemOta40aaSbaaSqaaiabdAgaMjabcYcaSiabdMgaPbqabaGccqWGNbWzdaWgaaWcbaGaemOyaiMaeiilaWIaemyAaKgabeaakiabcMcaPmaaCaaaleqabaGaeGOmaidaaaGcbaGaemOta40aaSbaaSqaaiabdAgaMjabcYcaSiabdMgaPbqabaGccqWGobGtdaWgaaWcbaGaem4zaCMaeiilaWIaemyAaKgabeaakiabcIcaOiabdAgaMnaaBaaaleaacqWGIbGycqGGSaalcqWGPbqAaeqaaOGaey4kaSIaem4zaC2aaSbaaSqaaiabdkgaIjabcYcaSiabdMgaPbqabaGccqGGPaqkaaaaleaacqWGIbGycqGH9aqpcqqGbbqqcqGGSaalcqqGdbWqcqGGSaalcqqGhbWrcqGGSaalcqqGubavaeqaniabggHiLdaaaa@6A6E@

where *N*_*f,i *_= ∑_*b*_*f*_*b,i *_and *N*_*g,i *_= ∑_*b*_*g*_*b,i *_are the sample sizes of the matrices columns at position *i*. If *χ*^2 ^exceeds the threshold of χth2
 MathType@MTEF@5@5@+=feaafiart1ev1aaatCvAUfKttLearuWrP9MDH5MBPbIqV92AaeXatLxBI9gBaebbnrfifHhDYfgasaacH8akY=wiFfYdH8Gipec8Eeeu0xXdbba9frFj0=OqFfea0dXdd9vqai=hGuQ8kuc9pgc9s8qqaq=dirpe0xb9q8qiLsFr0=vr0=vr0dc8meaabaqaciaacaGaaeqabaqabeGadaaakeaacqaHhpWydaqhaaWcbaGaeeiDaqNaeeiAaGgabaGaeGOmaidaaaaa@3248@ (*p *= 0.05) = 7.81 the null hypothesis that the base counts in both columns are from the same distribution is rejected with a p-value of 0.05. In order to simplify the analysis we simply count the number of significantly different positions. The example in Fig. 1 shows that for an appropriate alignment (with shift = 3) of the two matrices all *χ*^2^-values are below the χth2
 MathType@MTEF@5@5@+=feaafiart1ev1aaatCvAUfKttLearuWrP9MDH5MBPbIqV92AaeXatLxBI9gBaebbnrfifHhDYfgasaacH8akY=wiFfYdH8Gipec8Eeeu0xXdbba9frFj0=OqFfea0dXdd9vqai=hGuQ8kuc9pgc9s8qqaq=dirpe0xb9q8qiLsFr0=vr0=vr0dc8meaabaqaciaacaGaaeqabaqabeGadaaakeaacqaHhpWydaqhaaWcbaGaeeiDaqNaeeiAaGgabaGaeGOmaidaaaaa@3248@ threshold and hence no column appears to be different. Although the counts in some columns look quite different the limited sample size allows no statistically significant discrimination.

Obviously, the number of significantly different columns depends on the relative position of both matrices. In our algorithm we study all possible alignments with a minimum overlap of 6 bases and containing at least 75% of the information content of each matrix. We calculate the minimal number of different positions among these alignments. We call this number *D *and interpret it as the distance between the compared matrices. Fig. 1 illustrates that for a correct alignment of the ATF and CREB a distance *D *= 0 is obtained whereas other alignments lead to statistically significant different columns.

An advantage of the distance measure we use in comparison to earlier studies [15,17,19,20] is the emphasis on the limited sample size of many matrices. Only few binding sites, such as those recognized by the Sp1 factor, are characterized by hundreds of experimentally verified sites. The more common sample size is around 15–20 (see Tab. 1) and, thus, it is much more difficult to distinguish matrices. The *χ*^2 ^measure leading to the distance *D *takes into account the limited sample size in a statistically well defined manner. The proposed measure could be generalized by allowing gaps, using the sum of scores or by taking the number of possible shifts into account. Since we studied in this paper only rather strong similarities our simple discrete threshold *D *≤ 1 was sufficient.

### Correlation *C *between position frequency matrices scores

**Figure 2:**
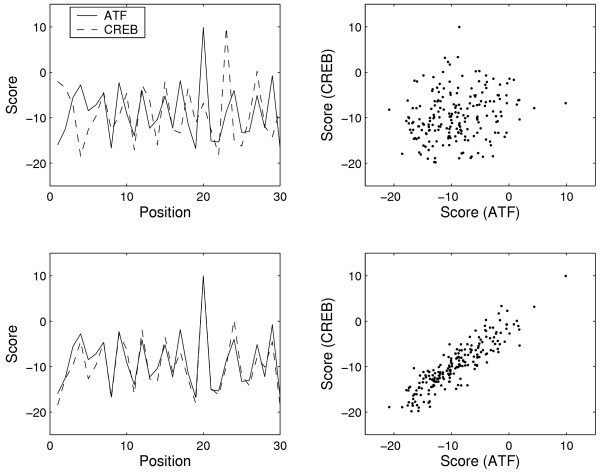
Comparison of ATF and CREB matrices: Correlation *C *of ATF and CREB scores along a test DNA sequence. Left: first 30 scores for ATF (solid line) and CREB (dashed line). Right: scores for ATF versus scores for CREB. Only the first 200 scores are plotted, but the full length of the test DNA sequence is 10000 bases. Upper (shift = 0): the matrices are not properly aligned (*C *= 0.068). Lower (shift = 3): the matrices ATF and CREB are properly aligned and both reveal a binding site at position 20 (*C *= 0.881).

The information on experimentally verified binding sites stored in PFMs can be exploited to predict novel sites. For this purpose position weight matrices (PWMs) can be constructed from the counts *f*_*b,i *_in the following manner [1,27]. First, the probability *p*_*b,i *_of a base *b *at a given position *i *is given by:

pb,i=fb,i+sbNi+∑b′=A,C,G,Tsb′
 MathType@MTEF@5@5@+=feaafiart1ev1aaatCvAUfKttLearuWrP9MDH5MBPbIqV92AaeXatLxBI9gBaebbnrfifHhDYfgasaacH8akY=wiFfYdH8Gipec8Eeeu0xXdbba9frFj0=OqFfea0dXdd9vqai=hGuQ8kuc9pgc9s8qqaq=dirpe0xb9q8qiLsFr0=vr0=vr0dc8meaabaqaciaacaGaaeqabaqabeGadaaakeaacqWGWbaCdaWgaaWcbaGaemOyaiMaeiilaWIaemyAaKgabeaakiabg2da9maalaaabaGaemOzay2aaSbaaSqaaiabdkgaIjabcYcaSiabdMgaPbqabaGccqGHRaWkcqWGZbWCdaWgaaWcbaGaemOyaigabeaaaOqaaiabd6eaonaaBaaaleaacqWGPbqAaeqaaOGaey4kaSYaaabeaeaacqWGZbWCdaWgaaWcbaGafmOyaiMbauaaaeqaaaqaaiqbdkgaIzaafaGaeyypa0JaeeyqaeKaeiilaWIaee4qamKaeiilaWIaee4raCKaeiilaWIaeeivaqfabeqdcqGHris5aaaaaaa@4D8D@

where *N*_*i *_= ∑_*b' *_*f*_*b',i *_denotes the sample size at the position *i *leading to the relative frequency fb,iNi
 MathType@MTEF@5@5@+=feaafiart1ev1aaatCvAUfKttLearuWrP9MDH5MBPbIqV92AaeXatLxBI9gBaebbnrfifHhDYfgasaacH8akY=wiFfYdH8Gipec8Eeeu0xXdbba9frFj0=OqFfea0dXdd9vqai=hGuQ8kuc9pgc9s8qqaq=dirpe0xb9q8qiLsFr0=vr0=vr0dc8meaabaqaciaacaGaaeqabaqabeGadaaakeaadaWcaaqaaiabdAgaMnaaBaaaleaacqWGIbGycqGGSaalcqWGPbqAaeqaaaGcbaGaemOta40aaSbaaSqaaiabdMgaPbqabaaaaaaa@347B@. This estimator is modified using pseudo-counts *s*_*b*_. As suggested earlier [28] we choose *sb *= Ni4
 MathType@MTEF@5@5@+=feaafiart1ev1aaatCvAUfKttLearuWrP9MDH5MBPbIqV92AaeXatLxBI9gBaebbnrfifHhDYfgasaacH8akY=wiFfYdH8Gipec8Eeeu0xXdbba9frFj0=OqFfea0dXdd9vqai=hGuQ8kuc9pgc9s8qqaq=dirpe0xb9q8qiLsFr0=vr0=vr0dc8meaabaqaciaacaGaaeqabaqabeGadaaakeaadaWcaaqaamaakaaabaGaemOta40aaSbaaSqaaiabdMgaPbqabaaabeaaaOqaaiabisda0aaaaaa@3078@, i.e. the pseudo-count is proportional to the standard deviation of the counted frequencies. Such a choice of relatively large pseudo-counts has a pronounced effect on PWMs with a small sample size. Due to the pseudo-counts the estimated probabilities are strictly positive even if zeros appear in the PFM. From the estimated probabilities *p*_*b,i *_we obtain the weights *w*_*b,i *_as follows:

wb,i=log⁡2pb,irb,
 MathType@MTEF@5@5@+=feaafiart1ev1aaatCvAUfKttLearuWrP9MDH5MBPbIqV92AaeXatLxBI9gBaebbnrfifHhDYfgasaacH8akY=wiFfYdH8Gipec8Eeeu0xXdbba9frFj0=OqFfea0dXdd9vqai=hGuQ8kuc9pgc9s8qqaq=dirpe0xb9q8qiLsFr0=vr0=vr0dc8meaabaqaciaacaGaaeqabaqabeGadaaakeaacqWG3bWDdaWgaaWcbaGaemOyaiMaeiilaWIaemyAaKgabeaakiabg2da9iGbcYgaSjabc+gaVjabcEgaNnaaBaaaleaacqaIYaGmaeqaaOWaaSaaaeaacqWGWbaCdaWgaaWcbaGaemOyaiMaeiilaWIaemyAaKgabeaaaOqaaiabdkhaYnaaBaaaleaacqWGIbGyaeqaaaaakiabcYcaSaaa@4134@

where *r*_*b *_refers to the *a priori *probability to find a base *b *in the DNA sequence. Consequently, the weights *w*_*b,i *_represent log-likelihood ratios to find a base *b *at a position *i*. Finally, the score *S*_*k *_around the position *k *of a test DNA sequence is a sum of the weights corresponding to bases observed in the DNA sequence at the subsequent positions starting from the position *k*. The sum *S*_*k *_is computed for each position *k *of the matrix along the DNA sequence. High positive scores *S*_*k *_indicate locations in the test DNA sequence with strong binding affinities whereas zero or negative scores are found elsewhere (Fig. 2).

This widely used technique of score calculation leads immediately to the second similarity measure (similar in spirit to the method used in [18], but modified to take into account the sample sizes of compared matrices). For two given matrices *f *and *g *we can directly obtain the corresponding scores Skf
 MathType@MTEF@5@5@+=feaafiart1ev1aaatCvAUfKttLearuWrP9MDH5MBPbIqV92AaeXatLxBI9gBaebbnrfifHhDYfgasaacH8akY=wiFfYdH8Gipec8Eeeu0xXdbba9frFj0=OqFfea0dXdd9vqai=hGuQ8kuc9pgc9s8qqaq=dirpe0xb9q8qiLsFr0=vr0=vr0dc8meaabaqaciaacaGaaeqabaqabeGadaaakeaacqWGtbWudaqhaaWcbaGaem4AaSgabaGaemOzaygaaaaa@30BC@ and Skf
 MathType@MTEF@5@5@+=feaafiart1ev1aaatCvAUfKttLearuWrP9MDH5MBPbIqV92AaeXatLxBI9gBaebbnrfifHhDYfgasaacH8akY=wiFfYdH8Gipec8Eeeu0xXdbba9frFj0=OqFfea0dXdd9vqai=hGuQ8kuc9pgc9s8qqaq=dirpe0xb9q8qiLsFr0=vr0=vr0dc8meaabaqaciaacaGaaeqabaqabeGadaaakeaacqWGtbWudaqhaaWcbaGaem4AaSgabaGaemOzaygaaaaa@30BC@ along all positions *k *in a given test DNA sequence. If the weight matrices are highly similar we expect positive peaks at nearly the same positions, i.e. a prediction of nearly the same set of binding sites. In order to quantify the similarity of both matrices we calculate the Pearson correlation coefficient along a test sequence. Here we also consider all possible relative shifts between two PWMs (with a minimum overlap of 6 bases) and then take the maximum correlation coefficient as the similarity measure *C *of the two matrices. We have found, that the correlation coefficients do not depend strongly on the value of the pseudo-counts and reflect mainly the relevant rare peaks.

In this paper we take as the test DNA sequence a random sequence with equidistributed bases. For specific applications it might be appropriate to use other test sequences such as upstream regions of the genes of interest.

### Sensitivity and specificity

Sensitivity and specificity of different methods for measuring similarities of profiles recognized by transcription factors were assessed as follows: since large sets of experimentally verified similar matrix pairs are not available, artificial sets were prepared. A representative initial matrix (either ATF or CREB) was resampled to construct a set of matrices. On average we probed the initial matrix 18 times (which corresponds to the median sample size of Transfac matrices). In order to study varying sample sizes for each generated matrix the number of samples was randomly chosen out of the range from 13 to 21. All the matrices generated this way should be classified as similar to each other. A set with matrices dissimilar to each other was prepared by random shuffling of the contents of the initial matrix. The nucleotide counts at each position were randomly reordered as well as the order of the positions. Additionally, we take into account different lengths of the matrices. Both sets were extended with random columns and the number of added columns was chosen randomly from zero to half of the length of the initial matrix. In the analysis, sensitivity was defined as the fraction of resampled matrices which were correctly identified as similar matrices. Specificity was defined as the fraction of random matrices which were identified as dissimilar. Six methods quantifying similarity of profiles were compared. The *D *(chi2th) and *C *(corr) functions were calculated as introduced above. Another score was defined as a sum of *χ*^2 ^obtained for each compared columns (chi2sum). Three other methods (introduced in [15,17,20]) calculate a total sum over all compared columns of: Euclidian distance (ned), column-column correlation (ccc) and scalar product of columns (sp).

## Results and discussion

In this paper two similarity measures of matrices are studied. The first quantifies for a given pair of matrices the number of significantly different columns *D*. The other represents the correlation *C *of binding sites scores along a DNA sequence for each of the given matrices.

### Comparison of both similarity measures

**Figure 3:**
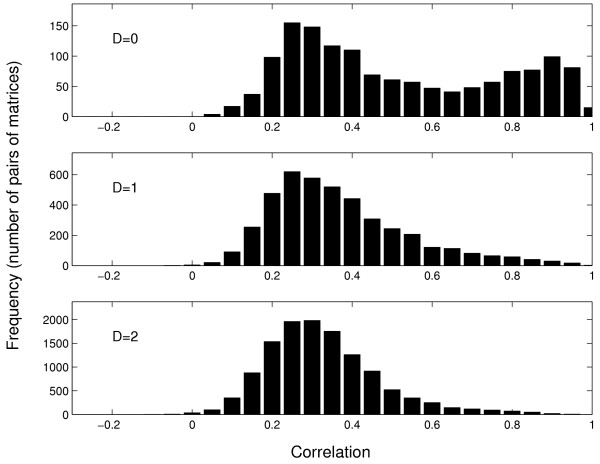
Combinations of both measures: Histograms of the correlation *C *of the scores vectors obtained for different values of the distance *D *(number of significantly different columns according to the *χ*^2 ^test). These data have been calculated for the Transfac matrices.

For the Transfac library we analyze whether the pairs of matrices with small distances *D *and high correlation coefficients *C *coincide, i.e. for what matrices the two measures give consistent results. Fig. 3 shows histograms of correlation coefficients *C *for matrices with distances *D *= 0, 1, 2. It turns out that there are many pairs of matrices with *D *= 0 and large values of *C *(see the right peak in the upper panel of Fig. 3). For such matrices the differences between their columns are negligible and predicted binding sites are essentially identical.

There are, however, also many pairs of matrices with *D *= 0 and relatively small correlation coefficients *C *(see the left peak in the upper panel of Fig. 3). These pairs refer mainly to matrices with a low information content and/or small sample size. In such cases the differences between columns are not statistically significant (many Ns in both consensus sequences) but their scores along a test DNA sequence correlate only weakly. For example, matrices V$STAT4_01 and V$MEF2_01 (see Transfac) are characterised by sample sizes *N *= 6, *N *= 5 respectively and have a distance *D *= 0 but a correlation *C *= 0.20.

There are also cases with a high correlation coefficient but with a distance *D *> 2. Such a situation appears for large matrices for which only a part is informative. For example matrices V$GR_01 and V$PR_01 (see Transfac) have a length of 27, but only six positions constitute the core sequence (TGTTCT). Among the others positions three are significantly different, leading to a distance *D *= 3 but these differences affect the correlation *C *only weakly (*C *= 0.92).

Several alternative measures have been proposed. We assessed the sensitivity and the specificity of these measures, as described in methods. The results of the comparison are presented in the supplemental Fig. 4. Both the our correlation measure and the column-to-column similarity give (for an appropriate threshold) a high specificity and sensitivity. However, in some cases, as illustrated above, adding a second criteria is useful to discard pairs involving large matrices for which only a part is informative. The *D *measure defined here can be used for this purpose. Both introduced measures quantify different properties and complement each other. Although alternative choices of measures might have been done, the advantage of using the correlation *C *is its implicit normalisation (the results do not depend much on the length and the sample size of the matrices) and the advantage of the distance *D *is its easy interpretation (number of different columns). Therefore, in the following, we focus on the most similar matrices based on the distance *D *and correlation *C *measures.

### Clusters of similar matrices

Here we study the matrices of both Jaspar and Transfac databases. We consider pairs of matrices for which *D *≤ 1 and *C *≥ 0.8 as highly similar. These stringent thresholds were chosen to identify the most obvious similarities and they imply that the matrices are almost indistinguishable from a statistical point of view and that their scores along DNA sequences are strongly correlated. We verified that for all these pairs of matrices both similarity measures select the same relative shift of the corresponding matrices.

**Figure 4:**
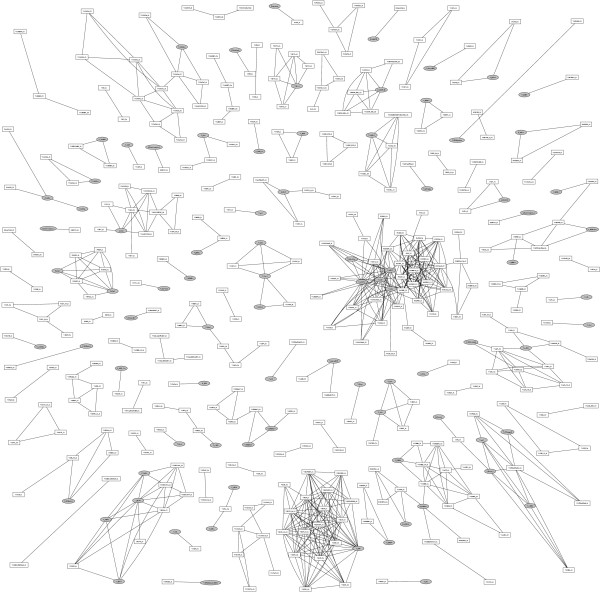
Graph showing similar matrices: Transfac matrices are indicated in white boxes, Jaspar matrices are indicated in gray ellipses. An edge is drawn between two matrices when *D *≤ 1 and *C *≥ 0.8. An enlarged version of this figure is available in the supplementary material (Fig. S1).

Fig. 4 shows an overview of all such matrices. Even though details of these clusters are only readable in the supplementary material (Fig. 1) the graph reveals interesting properties: The connecting lines visualizing high similarity join Jaspar matrices (ellipses) with Transfac matrices (boxes) in many cases. Consequently, our technique allows an automatic "alignment" of these collections of matrices. This is not a trivial task since the naming conventions used in the databases is different, and thus finding matrices corresponding to each other requires expert knowledge. We find that 84 matrices from Jaspar have counterparts in Transfac with *D *≤ 1 and *C *≥ 0.8. Another 16 matrices have somewhat smaller similarities *D *≤ 3 and *C *≥ 0.6. Only the Jaspar matrices P_HMG-1, P_HMG-IY and V_Ghlf, have no obvious "partners" in Transfac. A complete list of Transfac-Jaspar matrix pairs with high similarities is provided in the supplementary material (Tab. 1). Lists for other thresholds or other sets of matrices can be calculated through our web interface [29].

**Figure 5:**
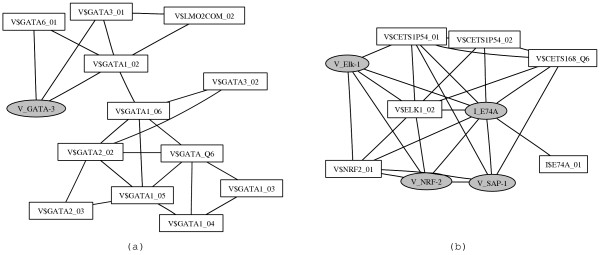
Clusters of similar matrices: Transcription factor families (a) GATA and (b) ETS.

In addition to the edges between Transfac and Jaspar matrices there are many clusters containing multiple Transfac or Jaspar matrices. These clusters reflect pronounced similarities in the matrix collections. There are for example, matrices of the same transcription factor with different degrees of stringency (see for instance AP1 matrices). Moreover, different transcription factors of certain families have almost identical binding motifs (see for example Myc-Max, USF and ARNT). A complete list of all clusters is provided in the supplementary material (Tab. S2). An interesting collection of structural classes of transcription factors has been compiled recently by Sandelin and Wasserman [17]. Consistent with their results we find also clusters of the ETS family (see cluster 2 in Tab. S2, also enlarged in Fig. 5b), bHLH transcription factors (cluster 15), and REL family (cluster 5).

In Fig. 5 we present enlargements of two selected clusters representing the GATA (panel a) and ETS (panel b) transcription factors family. The high similarity of these matrices cannot be directly noticed by inspection of names or consensus sequences. Furthermore, subgroups might be detected using our statistical approach. For example, the GATA cluster reveals that the Jaspar matrix has particularly high similarity to the Transfac entries GATA1_02, GATA3_01 and GATA6_01, but less similarities to other members of the GATA class. The clusters visualized in Fig. 4 and Fig. 5 can be exploited to reduce the number of matrices. Highly similar matrices match a DNA sequence either both or not at all. Therefore, one could construct "consensus matrices" as in [17] or one might select representative matrices in each cluster. In this way the number of overlapping predictions in the search for transcription factor binding sites can be decreased [17].

### Mapping of novel matrices to databases

A careful inspection of the clusters found automatically by our similarity analysis might reveal unexpected similarities pointing to possible cross-talks of different signaling cascades on the level of transcriptional regulation. As an example we discuss the regulation of circadian clock genes and cell cycle control [30,31]. In both processes bHLH transcription factors bind as dimers to E-boxes. The corresponding Myc-Max cluster appeared already in Fig. 4 (the largest cluster). In the mammalian circadian clock the CLOCK-BMAL1 dimer regulates clock genes such as *Per1*, *Per2*, *Per3*, *Cry1 *and *Cry2*. We found no matrix in Transfac or Jaspar describing explicitly the binding sites of CLOCK-BMAL1. Consequently, we constructed such matrices ourselves in two different ways. On one hand we collected 9 experimentally verified binding sites from 7 different clock genes [32-36]. On the other hand, we took from a SELEX experiment 10 sequences with high affinities to the CLOCK-BMAL1 dimer [37].

**Figure 6:**
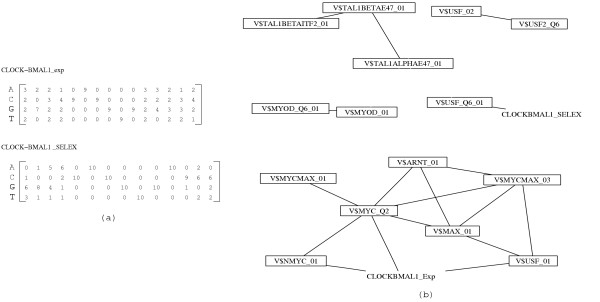
Mapping of CLOCK-BMAL1 matrices: (a) CLOCK-BMAL1 matrices based on experimentally characterised binding sites of clock genes and from a SELEX study (see Tab. S3 of the supplementary material for the list of these binding sites). (b) Mapping of CLOCK-BMAL1 matrices on E-box matrices. These matrices have been selected from the Transfac database and include MYC, MAX, ARNT, MYOD, USF, TAL1/E47 (see [35] for a review on E-box transcription factors). An edge is drawn when *D *≤ 1 and *C *≥ 0.8.

Both matrices are visualized in Fig. 6a. Details of the matrix construction are given in the supplementary material (Tab. S3). Both matrices contain the E-box consensus motif CACGTG but differ in the flanking regions.

Fig. 6b shows that these novel matrices have highly similar counterparts in Transfac (NMYC, MYC, USF). Consequently, cross-talk of the circadian clock with cell cycle regulation and tumor genesis can be expected at the level of transcriptional control. Indeed, the success of chronotherapies and recent detailed studies on cross-talk underline the dependence of circadian rhythms with tumor growth [38]. Also in the process of liver regeneration a pronounced effect of the circadian clock on cell cycle control has been found [39]. This example illustrates that a careful SELEX experiment combined with a mapping of the resulting matrix to known matrices can reveal possible functions of the corresponding transcription factor.

## Conclusion

Understanding gene regulation in higher eukaryotes is still challenging and current computational algorithms suffer from a large amount of false positive predictions [1,40]. In particular, mutually dependent position frequency matrices in databases such as Transfac or Jaspar lead to predictions of binding sites which overlap, what may be misinterpreted as a cluster of binding sites. Consequently, a careful pre-selection of matrices is essential. On one hand, expert knowledge can be used to select a subset of candidate matrices for the analysis of upstream regions. Such a selection is, however, subjective and novel combinations of transcription factor binding sites might be missed. On the other hand, for large scale computational studies, it is useful to have an automatic tool to detect similar matrices. Therefore, we introduce in this paper a method combining two independent similarity measures to compare position frequency matrices. This approach can be used to quantify similar matrices, to map the entries of different databases, and to cluster matrices.

The first similarity measure used in our approach is based on a *χ*^2 ^test. In contrast to earlier approaches based on normalized frequencies [15,17,20] we take into account the small sample size of many matrices. We count the number of significantly different matrix columns which defines the distance *D*. In this paper we focus on highly similar matrices with *D *≤ 1. In forthcoming studies the *χ*^2 ^measure might be taken directly to calculate distances of matrices in more detail.

The second measure is related to the primary application of position weight matrices – the prediction of binding sites in uncharacterized DNA sequences. We calculate for two matrices of interest the scores along a test DNA sequence and derive the Pearson correlation coefficient *C *of these vectors. Thus large values of *C *indicate that both matrices predict essentially the same binding sites. In this paper we take a 10000 bp long random sequence with equiprobable and independent bases as the test DNA sequence. However, the measure can be easily adapted also to other test sequences such as sets of promoter regions.

Our combined similarity measure was first used to map the Jaspar matrices to the Transfac database automatically. Then, requiring rather strong similarity (*D *≤ 1, *C *≥ 0.8) we identified similar matrices present in these databases and constructed clusters of almost indistinguishable matrices. By choosing only one representative matrix for each cluster it is possible to construct smaller sets of matrices as input of binding site prediction algorithms. Consequently, this approach decreases the number of overlapping binding site predictions. Moreover, such a reduced set constitutes a better input for methods predicting close occurrences of different binding sites (e.g. [16]). In order to eliminate false signals further, approaches such as phylogenetic footprinting [1,12,13], transcriptional profiling [14], ChIP on chip experiments [41,42] or modeling cis-regulatory modules need to be combined with a preselection of independent matrices. Our combined technique can be used to predict cross-talk on the level of transcriptional control. As an illustration we discuss the cluster of E-box binding bHLH transcription factors. Since circadian clock genes are regulated by a binding site quite similar to the Myc-Max motif, a strong interdependence of circadian regulation and cell cycle control is expected and is indeed known empirically for decades in connection with chronotherapies or liver regeneration.

Finally we use the similarity measures to assign newly derived matrices to known factors. To illustrate this application, we map an E-box matrix obtained from SELEX experiments with the CLOCK-BMAL1 dimer to the Myc-Max cluster. Thus the possible function of poorly characterized transcription factors can be predicted using affinity measurements combined with a comparison of the resulting matrix to database matrices.

## Availability

The method is available through a web interface at .

## Authors' contributions

SK, DG and HH designed the study. SK and DG were involved in programming and SK set up the web interface. SK, DG and HH interpreted the results and drafted the manuscript. All authors read and approved the final manuscript.

## Supplementary Material

Additional File 1 Correspondence between Jaspar and Transfac matrices: For each Jaspar matrix similar (*D *≤ 1 and *C *≥ 0.8) Transfac matrices are listed. 84 Jaspar matrices have at least one corresponding Transfac matrix. Click here for file

Additional File 2 Clusters of similar (*D *≤ 1 and *C *≥ 0.8) Jaspar and Transfac matrices. Click here for file

Additional File 3 Binding sites for Clock-Bmal1: Experimentally characterized binding sites for Clock-Bmal1 in clock genes and in selected sequences (SELEX experiment). Click here for file

Additional File 4 Comparison of different measures: specificity and sensitivity are determined as described in the "Methods" section of the paper for various thresholds of the different similarity measures. Specificity is defined as the fraction of the number of resampled matrices (TP on y-axis) found as similar. Sensitivity is defined as the fraction of the number of randomized matrices (TP on x-axis) found as dissimilar. Curves: "corr": correlation of scores along a DNA sequence, i.e. our score *C *(thresholds = 0.99, 0.95, 0.9, 0.8, 0.7...); "chi2th": our chi2 measure *D *(thresholds = 0, 1..8); "chi2sum": sum of column chi2 distances; "ned": normalized euclidian distance; "ccc": column-column correlation; "sp": column scalar product. Click here for file
