# Supplementary material for: Measuring similarities between transcription factor binding sites
Source: BMC Bioinformatics. 2005 Sep 28;6:237. doi: 10.1186/1471-2105-6-237 (PMC1261160; doi:10.1186/1471-2105-6-237)
Supplement: Additional File 1 — Correspondence between Jaspar and Transfac matrices: For each Jaspar matrix similar (D ≤ 1 and C ≥ 0.8) Transfac matrices are listed. 84 Jaspar matrices have at least one corresponding Transfac matrix. [file 1471-2105-6-237-S1.pdf]

Table S1. Correspondance between Jaspar and Transfac matrices. For each Jaspar matrix similar Transfac matrices ($D \leq 1$ and $C \geq 0.8$) are listed. 84 Jaspar matrice have at least one corresponding Transfac matrix.

JASPAR	TRANSFAC
V_HNF-1	V$HNF1_01, V$HNF1_Q6
V_NRF-2	V$ELK1_02, V$NRF2_01
V_c-ETS	V$PEA3_Q6
V_RREB-1	V$RREB1_01
P_Dof2	P$DOF1_01, P$DOF2_01, P$DOF3_01, P$PBF_01, V$PAX2_02
V_Hen-1	V$HEN1_02, V$HEN1_01, V$LBP1_Q6
V_HFH-2	V$FOXD3_01
V_E4BP4	V$CREBP1_01, V$E4BP4_01
X_NF-Y	VNFY_01, VALPHACP1_01, V$ALPHACP1_01
P_bZIP910	V$CREB_01, V$CREBP1CJUN_01, P$BZIP910_02, V$ATF6_01
V_SPI-B	V$PU1_Q6
V_p50	V$NFKAPPAB50_01
V_Pax6	V$PAX6_01
P_bZIP911	P$BZIP911_01, V$ATF6_01
V_SRF	V$SRF_01
V_SPI-1	V$PU1_Q6
V_Irf-2	V$IRF1_01, V$IRF2_01
V_p65	V$NFKAPPAB65_01, V$CREL_01, V$NFKB_Q6
V_Sox-5	V$SOX5_01, V$SRY_02, V$SOX9_B1
I_Dorsal_2	IDL_01, VNFKAPPAB65_01, V$CREL_01
V_Pbx	V$PBX1_02
V_n-MYC	V$NMYC_01, F$PHO4_01, V$MYCMAX_01, V$MAX_01, VUSF_01, VUSF_02, V$MYCMAX_02, V$SREBP1_01, V$ARNT_01, P$EMBP1_Q2, P$CPRF_Q2, $ARNT_02, V$MYCMAX_03, V$MYC_Q2
V_Irf-1	V$IRF1_01, V$IRF2_01, V$ICSBP_Q6
V_SP1	V$SP1_01
V_Max	V$NMYC_01, V$MAX_01, VUSF_01, VUSF_02, V$MYCMAX_02, V$ARNT_01, P$EMBP1_Q2, P$HBP1A_Q2, P$TAF1_Q2, P$CPRF2_Q2, V$MYCMAX_03, V$USF_Q6_01, VMYC_Q2, PCPRF2_01
V_SOX-9	V$SOX9_B1
V_HFH-1	V$HFH1_01
V_USF	V$NMYC_01, F$PHO4_01, V$MYCMAX_01, V$MAX_01, VUSF_01, VMYCMAX_02, V$SREBP1_01, V$ARNT_01, P$EMBP1_Q2, P$CPRF_Q2, V$ARNT_02, V$MYCMAX_03, VMYC_Q2, PTAF1_01
P_Dof3	P$DOF2_01, P$DOF3_01, P$PBF_01
V_CREB	V$CREB_01, V$CREB_02, V$CREB_Q4_01
V_AML-1	V$AML1_01, V$COREBINDINGFACTOR_Q6, V$AML1_Q6, V$AML_Q6
P_AGL3	P$AGL3_01, P$AGL3_02
I_CFI-USP	I$CF1_01, I$CF1_02
V_AP2alpha	V$AP2ALPHA_01
V_FREAC-2	V$FREAC2_01
V_PPARgamma	V$PPARG_02
V_p53	V$P53_01
P_Agamous	P$AG_01
I_Broad-complex_1	I$BRCZ1_01
V_deltaEF1	V$DELTAEF1_01, V$AREB6_02
V_Staf	V$STAF_02

JASPAR	TRANSFAC
V_Nkx	V$NKX25_01, V$NKX25_02
V_MEF2	V$RSRFC4_01
V_GATA-1	V$GATA1_01, V$GATA2_01
V_Ahr-ARNT	V$AHRARNT_01, V$AHR_Q5
I_Broad-complex_2	I$BRCZ2_01
V_MZF_1-4	V$MZF1_01
I_E74A	I$E74A_01, V$ELK1_02, V$CETS1P54_01, V$CETS1P54_02, V$NRF2_01, V$CETS168_Q6
V_RORalfa-1	V$RORA1_01, V$ERR1_Q2, V$ER_Q6_02
P_Athb-1	P$ATHB1_01, P$ATHB5_01
I_CF2-II	I$CF2II_01, I$CF2II_02
V_Elk-1	V$ELK1_02, V$CETS1P54_01, V$NRF2_01
V_HLF	VVBP_01, VHLF_01
X_TBP	V$TATA_01
V_SRY	V$SOX5_01, V$SRY_02
V_Myc-Max	V$MYCMAX_01, V$MYC_Q2
V_NF-kappaB	V$NFKAPPAB65_01, V$NFKAPPAB_01, V$NFKB_Q6
V_c-REL	V$NFKAPPAB65_01, V$CREL_01
V_COUP-TF	V$COUP_01, V$HNF4ALPHA_Q6, V$PPAR_DR1_Q2, V$HNF4_DR1_Q3, V$COUP_DR1_Q6
I_Snail	ISN_01, VE47_02, V$LMO2COM_01, V$E12_Q6, V$MYOD_Q6_01
I_Broad-complex_3	I$BRCZ3_01
V_Gfi	V$GFI1_01
V_GATA-3	V$GATA3_01, V$GATA1_02, V$GATA6_01
V_Chop-cEBP	V$CHOP_01
V_ARNT	V$NMYC_01, F$PHO4_01, V$MYCMAX_01, V$MAX_01, VUSF_01, VMYCMAX_02, V$ARNT_01, P$EMBP1_Q2, P$CPRF_Q2, P$TAF1_Q2, V$ARNT_02, V$MYCMAX_03, VMYC_Q2, PTAF1_01
V_HNF-3beta	V$HNF3B_01, V$HNF3ALPHA_Q6
I_SU_h	I$SUH_01
I_Dorsal_1	I$DL_01
V_PPARgamma-RXRal	V$PPARG_01
V_FREAC-4	V$XFD3_01, V$FREAC2_01
V_Thing1-E47	V$HAND1E47_01
V_RORalfa-2	V$RORA2_01
V_Yin-Yang	V$YY1_Q6
I_Hunchback	I$HB_01
V_c-MYB_1	V$CMYB_01
V_TCF11-MafG	V$AP1FJ_Q2, V$AP1_Q6
P_GAMYB	P$GAMYB_01
V_SAP-1	V$CETS1P54_01, V$NRF2_01, V$CETS168_Q6
V_Bsap	V$PAX5_01
V_HFH-3	V$HFH3_01
V_MZF_5-13	V$MZF1_02
V_Evi-1	V$EVI1_06, V$EVI1_01, V$EVI1_02, V$EVI1_03, V$EVI1_05
V_E2F	V$E2F_02, V$E2F_Q3, V$E2F_Q4, V$E2F_Q6, V$E2F1_Q3, V$E2F1_Q4, V$E2F1_Q6, V$E2F_03, V$E2F1DP1_01, V$E2F1DP2_01, V$E2F4DP1_01, V$E2F4DP2_01, V$E2F1DP1RB_01,V$E2F_Q3_01, V$E2F1_Q4_01, V$E2F1_Q6_01
V_c-FOS	V$AP1_Q6, V$AP1_Q4, V$BACH2_01, V$AP1_01
