# Supplementary material for: Measuring similarities between transcription factor binding sites
Source: BMC Bioinformatics. 2005 Sep 28;6:237. doi: 10.1186/1471-2105-6-237 (PMC1261160; doi:10.1186/1471-2105-6-237)
Supplement: Additional File 2 — Clusters of similar (D ≤ 1 and C ≥ 0.8) Jaspar and Transfac matrices. [file 1471-2105-6-237-S2.pdf]

Table S2. Clusters of similar ($D \leq 1$ and $C \geq 0.8$) Jaspar and Transfac matrices.

Cluster	Matrices
1	I$CF2II_02, I$CF2II_01, I_CF2-II
2	V$CETS1P54_01, V$ELK1_02, V$CETS1P54_02, V$NRF2_01, V$CETS168_Q6, V_NRF-2, I_E74A, I$E74A_01, V_Elk-1, V_SAP-1
3	V$CREB_01, V$ATF_01, V$CREBP1CJUN_01, V$VJUN_01, V$CREB_Q2, V$CREB_Q4, V$CREBP1_Q2, V$CREB_02, P$BZIP911_01, P$BZIP910_01, V$ATF6_01, V$ATF3_Q6, V$ATF4_Q2, V$CREB_Q4_01, P_bZIP910, P$BZIP910_02, P_bZIP911, V_CREB
4	V$E4BP4_01, V$CREBP1_01, V_E4BP4
5	V$CREL_01, V$NFKAPPAB65_01, V$NFKAPPAB_01, V$NFKB_Q6, V_p65, I_Dorsal_2, I$DL_01, V_NF-kappaB, V_c-REL, I_Dorsal_1
6	V$IRF2_01, V$IRF1_01, V_Irf-2, V_Irf-1, V$ICSBP_Q6
7	V$TAL1ALPHAE47_01, V$TAL1BETAE47_01, V$TAL1BETAITF2_01
8	V$HEN1_01, V$HEN1_02, V$AP4_Q6, V$AP4_Q5, V$LBP1_Q6, V_Hen-1
9	V$GATA2_01, V$GATA1_01, V_GATA-1
10	V$EVI1_02, V$EVI1_06, V$EVI1_03, V$EVI1_01, V$EVI1_05, V_Evi-1
11	V$CLOX_01, V$CDP_02
12	V$CDPCR3HD_01, V$CDPCR1_01
13	I$CF1_02, I$CF1_01, I_CFI-USP
14	V$CEBPB_02, V$CEBPA_01, V$CEBP_Q2, V$CEBP_Q2_01
15	VUSF_01, VMAX_01, V$ARNT_01, P$EMBP1_Q2, PGBP_Q6, PHBP1A_Q2, VUSF_Q6, PCPRF_Q2, V$NMYC_01, P$TAF1_Q2, P$CPRF3_Q2, P$CPRF2_Q2, P$O2_02, P$TGA1B_Q2, P$TGA1A_Q2, P$GBF_Q2, PABF_Q2, PABF1_01, P$O2_Q2, V$MYCMAX_03, P$RITA1_01, V$E4F1_Q6, P$HBP1B_Q6, V$USF2_Q6, VUSF_02, VMYC_Q2, F$PHO4_01, V$MYCMAX_01, P$HBPA1_Q6_01, P$ROM_Q2, P$TAF1_01, P$CPRF3_01, P$CPRF2_01, V_n-MYC, V$MYCMAX_02, V$SREBP1_01, V$ARNT_02, V_Max, V$USF_Q6_01, V_USF, V_Myc-Max, V_ARNT
16	V$GATA1_02, V$GATA3_01, V$GATA1_04, V$GATA1_03, V$LMO2COM_02, V$GATA1_05, V$GATA1_06, V$GATA2_02, V$GATA2_03, V$GATA3_02, V$GATA6_01, V$GATA_Q6, V_GATA-3
17	V$HNF3B_01, V$FOXD3_01, V$HFH3_01, V$HNF3ALPHA_Q6, V$FOX_Q2, V_HFH-2, V_HNF-3beta, V_HFH-3
18	IBCD_01, IDFD_01
19	V$HSF2_01, V$HSF1_01
20	VSRY_02, VSOX5_01, V_Sox-5, V$SOX9_B1, V_SOX-9, V_SRY
21	V$AP1_Q2, V$AP1FJ_Q2, V$AP1_Q6, V$AP1_Q4, V$BACH1_01, V$BACH2_01, V$AP1_01, V$AP1_Q2_01, V$AP1_Q6_01, V$AP1_Q4_01, V_TCF11-MafG, V_c-FOS
22	V$E2_Q6, V$E2_01
23	VHLF_01, VVBP_01, V_HLF
24	V$XFD2_01, I$CROC_01, V$FREAC2_01, V$FREAC7_01, V_FREAC-2, V_FREAC-4, V$XFD3_01
25	V$LMO2COM_01, V$MYOD_01, V$E12_Q6, V$E47_01, ISN_01, VMYOGENIN_Q6, VHEB_Q6, VAP4_Q6_01, V$MYOD_Q6_01, I_Snail, V$E47_02
26	V$TCF11_01, N$SKN1_01
27	V$HFH8_01, V$HFH1_01, V_HFH-1
28	VGEN_INI3_B, VGEN_INI2_B, V$GEN_INI_B
29	V$MINI19_B, V$MUSCLE_INI_B, V$MINI20_B
30	P$DOF2_01, P$DOF1_01, P$DOF3_01, P$PBF_01, V$PAX2_02, P_Dof2, P_Dof3
31	V$MMEF2_Q6, V$AMEF2_Q6, V$HMEF2_Q6, V$MEF2_01
32	V$HNF4_01_B, V$HNF4_01, V$PPARG_03, V$PPARA_01, V$DR1_Q3
33	V$AREB6_02, V$DELTAEF1_01, V_deltaEF1
34	V$MEIS1BHOXA9_01, V$MEIS1AHOXA9_01
35	V$E2F_Q3, V$E2F_02, V$E2F_Q4, V$E2F_Q6, V$E2F1_Q3, V$E2F1_Q4, V$E2F1_Q6, V$E2F_03, V$E2F1DP1_01, V$E2F1DP2_01, V$E2F4DP1_01, V$E2F4DP2_01, V$E2F1DP1RB_01, V$E2F_Q2, V$E2F_Q3_01, V$E2F1_Q4_01, V$E2F1_Q6_01, V_E2F

Cluster	Matrices
36	V$ZIC2_01, V$ZIC1_01, V$ZIC3_01
37	V$STAT5B_01, V$STAT5A_01
38	V$POU3F2_02, V$OCT1_07
39	V$FOXO1_01, V$FOXO4_01
40	N$DAF16_01, V$FOXO1_02, V$FOXO4_02, V$FOXO3_01
41	V$NKX22_01, V$NKX25_01, V_Nkx, V$NKX25_02
42	V$STAT6_01, V$STAT5A_03, V$STAT1_03, V$STAT3_02, V$STAT4_01, V$STAT5A_04, V$STAT6_02
43	P$ATHB5_01, P$ATHB1_01, P_Athb-1
44	V$ERR1_Q2, V$RORA1_01, VER_Q6_02, VT3R_01, V_RORalfa-1
45	V$HNF4ALPHA_Q6, V$COUP_01, V$PPAR_DR1_Q2, V$HNF4_DR1_Q3, V$COUP_DR1_Q6, V_COUP-TF
46	V$ALPHACP1_01, V$NFY_01, VNFY_Q6_01, VNFY_Q6, X_NF-Y
47	V$PAX8_01, V$PAX8_B
48	V$COREBINDINGFACTOR_Q6, V$AML1_01, V$AML1_Q6, V$AML_Q6, V_AML-1
49	F$ROX1_Q6, F$MAT1MC_02
50	V$P53_DECAMER_Q2, V$P53_02
51	VETS_Q4, VPEA3_Q6, V$TEL2_Q6, V_c-ETS
52	VIRF_Q6, VISRE_01
53	V$SREBP_Q3, V$SREBP1_02, V$SREBP1_Q6
54	V$HNF1_Q6, V$HNF1_01, V_HNF-1
55	VOCT_Q6, VOCT1_05, V$OCT1_Q5_01
56	V$HIF1_Q3, V$HIF1_Q5
57	P$MYBAS1_01, V$MYB_Q6, VMYB_Q5_01, PC1_Q2
58	V$E2A_Q2, V$MYOD_Q6
59	VSRF_Q4, VSRF_Q6, V$SRF_Q5_01
60	V$CREB_Q2_01, V$CREB_Q3
61	V$E2F_Q6_01, V$E2F_Q4_01
62	VGR_Q6_01, VGR_Q6
63	V$SP1_Q6_01, V$SP1_Q6, VETF_Q6, VSP1_Q4_01
64	I$ZESTE_Q2_01, I$ZESTE_Q2
65	V$NFAT_Q4_01, V$NFAT_Q6
66	VPR_02, VGR_01
67	V$DR4_Q2, V$LXR_DR4_Q3
68	V_RREB-1, V$RREB1_01
69	V_SPI-B, V$PU1_Q6, V_SPI-1
70	V_p50, V$NFKAPPAB50_01
71	V_Pax6, V$PAX6_01
72	V_SRF, V$SRF_01
73	V_Pbx, V$PBX1_02
74	V_SP1, V$SP1_01
75	P_AGL3, P$AGL3_01, P$AGL3_02, P_SQUA
76	V_AP2alpha, V$AP2ALPHA_01
77	V_PPARgamma, V$PPARG_02
78	V_p53, V$P53_01
79	P_Agamous, P$AG_01
80	I_Broad-complex_1, I$BRCZ1_01
81	V_Staf, V$STAF_02
82	V_MEF2, V$RSRFC4_01
83	V_Ahr-ARNT, V$AHRARNT_01, V$AHR_Q5
84	I_Broad-complex_2, I$BRCZ2_01
85	V_MZF_1-4, V$MZF1_01
86	X_TBP, V$TATA_01

Cluster	Matrices
87	I_Broad-complex_3, I$BRCZ3_01
88	V_Gfi, V$GFI1_01
89	V_Chop-cEBP, V$CHOP_01
90	I_SU_h, I$SUH_01
91	V_PPARgamma-RXRal, V$PPARG_01
92	V_Thing1-E47, V$HAND1E47_01
93	V_RORalfa-2, V$RORA2_01
94	V_Yin-Yang, V$YY1_Q6
95	I_Hunchback, I$HB_01
96	V_c-MYB_1, V$CMYB_01
97	P_GAMYB, P$GAMYB_01
98	V_Bsap, V$PAX5_01
99	V_MZF_5-13, V$MZF1_02
