# Supplementary material for: Measuring similarities between transcription factor binding sites
Source: BMC Bioinformatics. 2005 Sep 28;6:237. doi: 10.1186/1471-2105-6-237 (PMC1261160; doi:10.1186/1471-2105-6-237)
Supplement: Additional File 3 — Binding sites for Clock-Bmal1: Experimentally characterized binding sites for Clock-Bmal1 in clock genes and in selected sequences (SELEX experiment). [file 1471-2105-6-237-S3.pdf]

Table S3. Experimentally characterized binding sites for Clock-Bmal1 in clock genes and in selected sequences (SELEX experiment):

Exp. Charact. Gene	Binding site	Organism	Reference
mper1	tttagcCACGTGacagtg	mouse	Gekakis et al, 1998
mper1	taagcaCACGTGggccct	mouse	Gekakis et al, 1998
mper1	caagtcCACGTGcaggga	mouse	Gekakis et al, 1998
per	tcggctCACGTGaatca	Drosophila	Darlington et al, 1998
tim	gccgctCACGTGgcgaac	Drosophila	Darlington et al, 1998
AVP	cacgccCACGTGtgtccc	mouse	Munoz et al, 2002
CYC	gcagacCACGTGagagcc	human	Munoz et al, 2002
zfper4	gaagCACGTGtactcg	zebrafish	Vallone et al, 2004
AANAT	tatgtgCACGTGcggtgc	rat	Chen et al, 2000

Selected sequences	Consensus binding site	Organism	Reference
sequence 1	ggggCACGTGacac	-	Hogenesch et al, 1998
sequence 2	ggtaCACGTGaccc	-	Hogenesch et al, 1998
sequence 3	tgaaCACGTGaccc	-	Hogenesch et al, 1998
sequence 4	tgaaCACGTGactc	-	Hogenesch et al, 1998
sequence 5	gggcCACGTGacct	-	Hogenesch et al, 1998
sequence 6	gggaCACGTGaccg	-	Hogenesch et al, 1998
sequence 7	ctaaCACGTGaccg	-	Hogenesch et al, 1998
sequence 8	gaacCACGTGagct	-	Hogenesch et al, 1998
sequence 9	tgaaCACGTGacac	-	Hogenesch et al, 1998
sequence 10	gggtCACGTGactc	-	Hogenesch et al, 1998
